# Coronavirus Disease 2019 and the Case to Cover Undocumented Immigrants in California

**DOI:** 10.1089/heq.2020.0049

**Published:** 2020-11-25

**Authors:** Diana L. Torres-Pinzon, Walter Solorzano, Sue E. Kim, Michael R. Cousineau

**Affiliations:** ^1^ Keck School of Medicine, University of Southern California, Los Angeles, California, USA.; ^2^ Department of Preventive Medicine, Keck School of Medicine, University of Southern California, Los Angeles, California, USA.

**Keywords:** immigrant health, health policy, COVID-19

## Abstract

Latinos have been affected at higher rates in California. These include undocumented immigrants who are the largest group of Californians that remains uninsured. This population has limited access to health care services and coverage options. The coronavirus disease 2019 (COVID-19) pandemic has shown that undocumented immigrants are particularly vulnerable during this outbreak as they are more likely to delay seeking medical care and lack a regular source of care. In addition, many work in essential services, have low or moderate incomes, and live in overcrowded conditions increasing their risk for exposure to COVID-19. To reduce the state's COVID-19 burden, California should expand comprehensive Medi-Cal to all eligible individuals regardless of immigration status.

## Introduction

There were >1 million coronavirus disease 2019 (COVID-19) cases in California and >18,000 deaths as of November 17, 2020 (Ref.^1^). Latinos are disproportionately affected by COVID-19 accounting for 60% of cases in California, whereas comprising 39% of the state's population (Table 1).^1^ Yet, efforts to focus resources to reduce the impact of the pandemic has been hindered by long-standing federal policies restricting access to health care for immigrants, particularly those who are undocumented.^2–4^

**Table 1.** The Percentage of Coronavirus Disease 2019 Cases and Associated Deaths in California by Race and Ethnicity

Race/ethnicity	Cases,^*^ % (n), (836,606 total cases)	Death cases,^**^ % (n), (16,298 total deaths)	Race and ethnicity distribution of the California 2019 Population (total 39.51 million)
Latino	60.0	48.5	38.9
White	18.4	30.3	36.6
Asian	5.6	11.7	15.4
African American/Black	4.3	7.4	6.0
Multirace	1.2	0.8	0.3
Native Hawaiian and other Pacific Islander	0.5	0.5	0.5
American Indian or Alaska Native	0.3	0.3	2.2

Data obtained from the California Department of Public Health as of November 17, 2020 (Ref.^1^).

^*^ 294,199 (30%) missing race/ethnicity.

^**^ 164 (1%) missing race/ethnicity.

Although the barriers faced by the undocumented immigrant population are found throughout the nation, the challenge is particularly acute in California where a quarter of the population is foreign born.^5^ Undocumented immigrants make up about 6% or 2.4 million of the California's population.^6^ Most are low-income Latinos, between the ages of 26 and 64 years.^7^ Many work low paying jobs, experience significant financial instability, are rent-burdened and food-insecure.^8^ Still, undocumented immigrants contribute an estimated $3 billion in federal state and local taxes each year, including payments to Social Security, Medicare and Medicaid.^9,10^

Undocumented immigrants face significant obstacles to needed health care. Although many undocumented immigrants are employed, many are not covered by employer-sponsored health insurance. In addition, undocumented immigrants have limited access to federal health insurance programs.^11^ For example, the Deficit Reduction Act of 2005 made legal immigration status a requirement when applying for or renewing full-scope Medicaid coverage (health insurance for low income uninsured).^12^ For Medicare, health coverage for those >65 years, undocumented immigrants are simply ineligible.

Furthermore, the Patient Protection and Affordable Care Act (PPACA) explicitly excluded undocumented immigrants from all subsidies and expansions.^13^ The PPACA also restricted several provisions for legally authorized immigrants with <5 years of residence in the country.^11^ Thus, although uninsured rates have declined nationally, 45% of nonelderly undocumented immigrants remain uninsured, approximately twice the percentage as immigrants with legal documentation.^14^

In California as in other states, undocumented immigrants comprise the largest group of the residually uninsured, accounting for 37% of the state's uninsured population.^15^ Before the PPACA, only 20% of the state's undocumented immigrants received job-based coverage compared with 68% of citizens.^16^ This has changed little since PPACA implementation in California. Because of limited access to federally funded health programs, California had taken several steps to expand coverage to this population, including children and young adults.^17^ California offers restricted-scope Medi-Cal for those who are ineligible for full benefits due to their immigration status. This covers pregnancy-related services, emergency services, and state-funded long-term care.^18^

The Deferred Action for Childhood Arrivals (DACA) recipients are eligible for full-scope Medi-Cal although paid entirely with state funds.^18^ In 2002, California expanded the Children's Health Insurance Program to include prenatal care for eligible undocumented women.^19^ Then in 2015, Senate Bill 75 expanded full-scope Medi-Cal, using state-only funds, to low-income children <19 years regardless of immigration status.^18,20,21^ Later in 2019, SB-104 expanded coverage to low-income undocumented young adults between the ages of 19 and 25 years.^22^ In addition to state programs, several counties have implemented programs that provide limited scope of services to undocumented adults. These vary by county in the scope of care available and income and health-related criteria for eligibility.^23^ Some counties offer no program at all.^23^

## Impact of COVID-19 on the Undocumented Population

Limited coverage options for undocumented immigrants are important because being uninsured against health problems creates financial barriers to needed health care. Uninsured individuals are less likely to have a regular source of care and are more likely to delay seeking primary and preventive care services.^24,25^ This contributes to presentation with advanced illnesses, multiple comorbidities, poor treatment management, and overall poor health outcomes. Yet COVID-19 highlights the importance of timely access to health care and the urgent need for covering all the uninsured, including immigrants, who have been left behind.

However, despite the disproportionately high impact on Latinos, barriers to needed care have become more pronounced for undocumented immigrants in the wake of the COVID-19 pandemic. If these individuals suddenly experience COVID-19 symptoms or are told by contact tracers they should seek care, many will not know how or where to get medical assistance.^26^ Although telehealth visits have expanded due to social distancing, stay at-home orders and more flexible reimbursement policies, these services are not available to patients without adequate access to technology.^26^ Limited telehealth options disproportionately affect low-income families, the uninsured, and those with low English proficiency.

Without timely access to primary care services, undocumented immigrants may delay seeking care and opt for costlier emergency services after the disease has progressed to a more dangerous level. Others may forgo care altogether possibly risking death and exposing others to the virus.

This also increases the risk of complications from other chronic diseases as well from COVID-19. Lack of resources and fear of COVID-19 may also discourage people from seeking care for the management and treatment of other medical conditions. Health provider are concerned about a decline in emergency department visits and office visits for prevention, primary, and specialty care. Sick people may delay or forgo care for poorly controlled chronic diseases, cancer treatment, or medication checks. Many may defer medical therapy or surgical procedures. Others are unable to afford medications since they face more economic hardships due to the pandemic.

Many immigrants are hesitant to seek medical care due to the new public charge regulations. These rules allow officials to consider the use of public benefits as a reason to deny requests for legal permanent resident status or admission to the country.^27^ Under the new regulations, additional public benefits would be considered for public charge determination, including nonemergency Medicaid for nonpregnant adults and the Supplemental Nutrition Assistance Program.^27^ Fear of compromising resident status could discourage immigrants from using medical services.^28^ In response to this and to ensure that immigrant families seek needed services, the U.S. Citizenship and Immigration Services announced that health services related to COVID-19 will not be considered a public charge.^14^ Although this is encouraging for immigrants in need of COVID-19 health services, it may not be enough to counteract the mistrust and fear created by years of anti-immigrant rhetoric.

The federal government has enacted a series of stimulus bills to counteract the economic impact of the pandemic. However, these programs categorically exclude undocumented immigrants. Most prominent was the Coronavirus Aid, Relief, and the Economic Security Act that provided direct payments of up to $1200 for single filers, $2400 for joint filers, and an additional $500 per claimed child in the 2019 fiscal year.^2^ Federal law, however, prohibits undocumented immigrants from receiving these benefits, including those who are married to undocumented immigrants and 5.1 million children who are U.S. citizens.^29,30^

Because many immigrants are ineligible for federal assistance, California created the Disaster Relief Fund that allocates $75 million for undocumented immigrants with additional funds from philanthropy.^31^ An estimated 150,000 undocumented adults will benefit from this fund with a one-time payment of $500 and maximum of $1,000 per household.^31^ In addition, California provides COVID-19 testing and treatment through Medi-Cal emergency services.^32^ Although undocumented immigrants have not benefited from the federal relief packages, many are critical parts of the United States' response to the COVID-19 pandemic. Many work in essential services such as health care, cleaning services, agriculture, manufacturing, or are part of the food distribution supply chains.^26,33^ Many farmworkers are immigrants and about half are undocumented.^34^ They work in the fields not only in the California's central valley but also in counties along the central coast and the borders with Mexico, Oregon, Arizona, and Nevada. These workers are essential to managing 13% of the entire national agricultural value.^35^ Yet, they are at increased risk of exposure and poor health outcomes due to inadequate protective measures, limited access to health care services, underlying medical conditions, and poor working conditions.^34,36^

Many immigrants have lost their jobs due to the pandemic but are not eligible for unemployment benefits.^37^ Those who have held onto their jobs have minimal or no paid sick leave forcing workers to choose between taking unpaid time to see a physician for themselves or their family members, or put food on the table or pay the rent. All of these exacerbate the financial strain on low-income immigrant families, including their citizen children that existed even before the pandemic.^14^ Many immigrants have jobs that require them to be on-site, for example, those who work in agriculture, cleaning services, or as factory workers. Many cannot follow stay-at-home orders that increase the chance of exposure to the coronavirus at the workplace or in traveling to and from their jobs.

Undocumented immigrant families often reside in dense low-income urban communities, and crowded apartments or homes often with multiple families and generations in a rental unit.^26^ This makes it difficult to follow social distancing guidelines increasing the risk of encountering or transmitting the coronavirus to others in the home.^26^ Still others may have come here from U.S. Immigration and Customs Enforcement (ICE) detention centers where they may be exposed to the coronavirus while waiting in congregate and crowded conditions awaiting hearings or meetings with attorneys.

For the past few years, California has tried to close the coverage gap. First, the state provided full-scope coverage to undocumented children under SB-75.^20^ The Legislature continued to try to bring other populations into Medi-Cal. Previously unsuccessful bills (AB-4 and SB-29) were reintroduced calling for the expansion of full-scope Medi-Cal to all low-income individuals, regardless of immigration status.^38,39^ However, the cost of covering all adults particularly the working age population had kept the Governor from approving this expansion. In its final form, SB-29 called for expansion of full-scope Medi-Cal to undocumented seniors ages 65 years and older.^39^ The 2020–2021 budget initially proposed $80.5 million toward Medi-Cal expansion for an estimated 27,000 undocumented seniors.^40^ However, these expansions have been suspended because of the pandemic-related economic crisis facing California.^40^

## Conclusion

Undocumented immigrants have been disproportionately affected by COVID-19. However, many faced barriers to needed health care before the COVID-19 pandemic. Now with so many affected by or at-risk for COVID-19, undocumented immigrants will face additional barriers to testing and access to treatment, follow-up and preventive care, posing significant public health challenges going forward. Moreover, faced with barriers to care and declining incomes, many will delay seeking care for other health problems or forgo important preventive services, including childhood vaccinations.

California could enact these changes without federal approval but like nearly all states, California is facing unprecedented budget shortfalls with the economy declining in response to the pandemic. Although the short-term prospect of covering all undocumented immigrant is quite limited given California's budget crisis, the state should move forward as soon as possible to expand full-scope Medi-Cal and other insurance opportunities for the ∼1.4 million low-income undocumented immigrants who reside in the state.^17^

These measures could include working with employers, the agricultural industry, entertainment, and hospitality, all of which will depend on a healthy workforce as they recover from the pandemic-induced recession.

Expanding health coverage to the undocumented would provide better access for those affected by COVID-19 who would otherwise be unable to obtain needed health care because of their immigration status and inability to pay on the private market. Health coverage can reduce hesitancy to seek testing and medical care in case of suspected COVID-19 exposure or onset of symptoms. This in turn would help prevent the spread of the virus by encouraging isolation measures and providing timely medical care. Moreover, by expanding comprehensive Medi-Cal to all undocumented individuals, the state has the potential to lower transmission rates and mortality rates associated with COVID-19 for all Californians and help the state restore its once vibrant economy.

Although this study focuses on California, the benefits to undocumented immigrants will apply to most states especially those with large immigrant populations. These include Texas (1.6 million), Florida (825,000), New York (650,000), New Jersey (450,000), and Illinois (425,000).^41^ Expanding coverage in these states, as well as the nation would yield similar benefits to improving the health of all residents. Obtaining primary and preventive care is important not just to immigrant families themselves but also critical to the nation's broader COVID-19 pandemic response, justifying a national policy effort to cover all people regardless of immigration or documentation status.

## Abbreviations Used

COVID-19: coronavirus disease 2019
PPACA: Patient Protection and Affordable Care Act
